# The Anticholinesterase Phenserine and Its Enantiomer Posiphen as 5′Untranslated-Region-Directed Translation Blockers of the Parkinson's Alpha Synuclein Expression

**DOI:** 10.1155/2012/142372

**Published:** 2012-05-29

**Authors:** Sohan Mikkilineni, Ippolita Cantuti-Castelvetri, Catherine M. Cahill, Amelie Balliedier, Nigel H. Greig, Jack T. Rogers

**Affiliations:** ^1^Neurochemistry Laboratory, Massachusetts General Hospital (East), CNY2, 149, 13th Street, Charlestown, MA 02129, USA; ^2^MIND, Massachusetts General Hospital, Charlestown, MA 02129, USA; ^3^Drug Design and Development Section, Laboratory of Neurosciences, Intramural Research Program, National Institute on Aging, Baltimore, MD 21224, USA

## Abstract

There is compelling support for limiting expression of alpha-synuclein (*α*-*syn*) in the brains of Parkinson's disease (PD) patients. An increase of *SNCA* gene copy number can genetically cause familial PD where increased dose of this pathogenic protein correlates with severity of symptoms (triplication of the *SNCA* gene causes dementia in PD patients). Gene promoter polymorphisms were shown to increase *α*-synuclein expression as a risk for PD. Cholinesterase inhibitors can clinically slow cognitive decline in the later stages of PD etiology similar to their widespread use in Alzheimer's disease (AD). Pertinent to this, we identified that the well-tolerated anticholinesterase, phenserine, blocked neural *SNCA* mRNA translation and tested for targeting via its 5′untranslated region (5′UTR) in a manner similar to its action to limit the expression of the AD-specific amyloid precursor protein (APP). Posiphen, its better-tolerated (+) enantiomer (devoid of anticholinesterase action), repressed neural *α*-synuclein translation. Primary metabolic analogs of posiphen were, likewise, characterized using primary fetal neurons grown *ex vivo* from the brains of Parkinson's transgenic mice expressing the human *SNCA* gene.

## 1. Introduction

Parkinson disease (PD) is a slowly progressive neurodegenerative disease affecting up to 3% of the population over the age of 65 years. Clinically, it is characterized by a set of motor symptoms and nonmotor symptoms that can include dementia. Motor symptoms include rigidity, postural instability, bradykinesia, and resting tremor [1, 2]. The core pathological feature correlating with most of these motor symptoms is a loss of dopaminergic neurons from the substantia nigra pars compacta (SNc). Pathological inclusions, known as Lewy bodies, are found within some of the remaining dopaminergic neurons [3]. The destruction of the dopaminergic neurons at onset is at least 70% and, at the end stage of the disease the loss of dopamine (DA) neurons, can exceed 95% [3]. Therapy for the symptoms of PD is based primarily on replacement of dopaminergic function and can be remarkably effective in alleviating motor symptoms for a number of years [1, 4]. Additional nonmotor symptoms can be present and include depression, anxiety, sensory abnormalities, anosmia, sleep, and autonomic disorders, in addition to dementia [1, 5–8].

Particular nonmotor symptoms, like sleep disturbances [9], loss of smell [6, 10], and depression, can occur well before the presence of detectable motor symptoms [5]. The presence of psychotic symptoms, which can affect up to 40% of the patients, is often associated with anti-parkinsonian treatment and is dose dependent [2, 7]. By contrast, the neuropsychiatric symptoms of PD, like depression and dementia, are more common at the later stages of the disease [6, 8, 11, 12]. These neuropsychiatric symptoms may be correlated directly to the progression of PD itself as in PD dementia or PDD, to the presence of Dementia with Lewy Bodies (DLB) or to the presence of comorbidities like Alzheimer's Disease (PD/AD) [2]. The incidence of dementia in PD is six times higher than in age-matched controls; in all forms of dementia associated with PD, the use of cholinesterase inhibitors may be beneficial [2, 13–17].

The etiology of PD is still elusive [18]. A variety of genetic factors have been linked to the etiology of familial PD [19]. The first was the protein alpha-synuclein (*α-syn*), an abundant brain protein that appears to be involved in vesicle trafficking and participates in the regulation of DA release [20]. The first reports to implicate *α-*syn in PD described mutations in the protein, which cause autosomal dominant forms of the disease [21, 22]. Subsequent studies revealed that such mutations were exceedingly rare but also that aggregates of  *α-syn* could be found in all cases of familial and sporadic PD [23]. It is important to recognize that the genetic mechanisms identified to date are individually rare and, collectively, represent only a small fraction of the cases of PD observed by practitioners. This hence leaves the vast majority of PD unexplained at present.

Events associated with the inflammatory cascade [24] as well as with iron metabolism [25, 26] and translational control of gene expression have been modeled to be associated with PD and LBD [27]. There are also reported examples of disrupted signaling events such as occuring in response to inflammation cytokines: for example, mutations to the signaling kinase LRRK-2 may activate inflammatory events in this neurodegenerative disease [24, 28], perhaps affecting novel signalling pathways [24]. Certainly, increased iron in the individual dopaminergic neurons of the substantia nigra (SN) has been reported to be closely associated with the pathogenesis of PD [29], and antioxidants have been tested as a means to alleviate the severity of PD [30]. DLB brains exhibit lowered* SNCA* mRNA but higher insoluble protein, suggesting misregulation of *SNCA* mRNA translation additional to its clearance by chaperones [31], although reduced autophagy may provide the explanation. Translational control of  *α-syn* may be governed, at least in part, by a uniquely configured 5′untranslated region (5′UTR) of its transcript, which encodes a homology with the known APP and ferritin iron-responsive elements [32–35].

*α*-Synuclein is, in fact, the central ~15 kDa protein implicated in the pathogenesis of neurodegenerative  *α*-synucleinopathies [36], including PD—the most prevalent movement disorder in humans. Other alpha synucleinopathies include DLB, Lewy body variant of Alzheimer's disease (AD), and multiple system atrophy. In these disorders,  *α-syn *undergoes a conformational change and oligomerization, causing a toxic gain of function. Neurodegenerative deposition of  *α-syn* aggregates occurs, most commonly in Lewy bodies. Lewy bodies are rare in PD patients, present only in the surviving neurons.  *α-syn* aggregates are in dystrophic neurites. Glial cytoplasmic inclusions are in multiple systems atrophy [37]. Pathogenic pathways of  *α*-synuclein and amyloid-*β* (Aß) converge via interaction of these two amyloidogenic proteins, which coprecipitate into ß-pleated oligomers and insoluble fibrils [38–40], although Aß and  *α-syn *rarely colocalize in brain amyloid deposits [41]. There is certainly also overlap between the 5′UTRs of the *SNCA *and amyloid precursor protein (APP) transcripts, another similarity that provides a further link between PD and AD [27, 33–35].

Such overlap in the 5′UTRs of *SNCA *and APP mRNAs is consistent with our and other groups' recent demonstrations that both are key players in iron metabolism and  *α-syn *may be translationally controlled by cellular iron levels as was demonstrated for APP expression [35, 42]. In this regard, APP is a copper-dependent iron export ferroxidase [43], and  *α-syn* a Cu-binding ferrireductase [44, 45]. These similarities, together with the utility of anticholinesterases in AD and PD, provided our rationale to test whether known anticholinesterases that are beneficial and A*β* lowering [46] would, likewise, provide anti-*α-syn* activity in neural cells.

The most common therapy for PD is L-dopa that is routinely used to overcome the archetypical problems of tremor. As disease progresses, however, clinically available anticholinesterases have been increasingly empirically used, based in part on an putative impairment of the cholinergic system in developing PD [47].

Therefore, our study investigated the effectiveness of the cholinesterase inhibitor phenserine and its noncholinergic (+) chiral enantiomer, posiphen in lowering  *α*-synuclein expression in neural cell lines. Both agents have been shown to effectively lower APP synthesis rate both in neuronal cultures and animals via translational regulation mediated at the level of the APP mRNA 5′UTR. As both agents have been developed through to clinical studies and appear well tolerated in AD, if effective in reducing  *α-syn* levels, they hold translational promise as potential therapeutics for PD.

 Furthermore, as posiphen can be effectively dosed in rodents and humans in far greater amounts than phenserine, thereby generating high levels of its primary metabolites, the three most prominent primary metabolic products of posiphen were also characterized. As a model to examine the  *α-syn*-lowering efficacy of these agents, we assessed the cellular therapeutic impact of these screened *SNCA *5′UTR-directed translation blockers to reduce  *α-synuclein *expression in neural cells lines and subsequently also in the primary neurons from the PAC/Tg(*SNCA*) genomic human *SNCA *mouse model of PD (Figures 2 and 6) [48].

### 1.1. Hypothesis/Model

Our model to be tested is shown in Figure 7. The overarching hypothesis is that the drug phenserine (anticholinesterase) and its enantiomer are known translation blockers of the amyloid precursor protein of Alzheimer's disease (AD) and, as such, have been tested in clinical trials for their antiamyloid efficacy and potential to improve cognition. We noted that the RNA target in the 5′UTR of APP mRNA was similar to that found in the alpha-synuclein transcript. Therefore, we decided to see if posiphen and phenserine might block  *α*-synuclein, the central culprit protein in PD. To achieve this end, we conducted Western Blot experiments firstly with SH-SY5Y dopaminergic neuroblastoma cells and then with primary E18 neurons from a genomic mouse model of PD. Our rationale for conducting this study was to determine if posiphen and phenserine are two 5′UTR-directed drugs that would reduce alpha-synuclein expression to provide therapeutic benefit to Parkinson's disease patients.

## 2. Materials and Methods

### 2.1. Materials

 Dulbecco's modified essential medium (DMEM, catalog no. 12-614Q); FBS (catalog no. 14-503E), L-glutamine (catalog no. 17-605E), penicillin/streptomycin (catalog no. 17-602E); phenol red free media (phenol red-free DMEM with 4.5 g/L D-glucose (catalog no. 12-917F)) were purchased from Lonza (Portsmouth, NH). Trypsin/EDTA (catalog no. 25-053-Cl) was purchased from Cellgro. Steady-Glo (catalog no. E2250) was purchased from Promega (Madison, WI). For the Western Blot secondary screen, penicillin/streptomycin was acquired from Bio-Whittaker (Walkersville, MD), mouse monoclonal anti-alpha-synuclein was purchased from BD Transduction Laboratories, and anti-beta-actin was acquired from Chemicon, Inc. We routinely use 384-well plates (catalog no. 3570), which were purchased from Corning and Falcon TC flasks (catalog no. 353112) were purchased from Becton Dickinson (Waltham, MA). SH-SY5Y cells were purchased from the ATCC, Manassas, VA.

### 2.2. Cell Culture

 Transfected SH-SY5Y neuroblastoma cells were grown to confluency in 35 mL complete DMEM with 4.5 g/L D-glucose supplemented with 10% FBS, 200 *μ*M L-glutamine, 100 *μ*M penicillin/streptomycin, and 200 *μ*g/mL geneticin in a T175 TC flask in a TC incubator (37°C, 95% humidity, 5% CO_2_) (doubling time = 24 h). Untransfected SH-SY5Y counterparts were grown in the absence of geneticin. Cells were harvested by washing the monolayer quickly with 5 mL trypsin/EDTA (1X), aspirating, then adding 5 mL trypsin/EDTA and incubating for 5 min at 37°C, 95% humidity, 5% CO_2_, after which cells were collected into phosphate-buffered saline and centrifuged into a pellet for storage at −70°C.

 Primary cortical neurons from wild-type mice and from the PAC-Tg(*SNCA*(wt) human *SNCA *genomic mice [48] were cultured as outlined by the method of Ray et al., 2009 [49]. Briefly, we recovered the embryonic day 15–18 pups after sacrificing pregnant females, separated out the brain, and removed the meninges and blood vessels. We then dissected out the cortices and placed them in separate eppendorf tubes containing 500 uL of HBSS without Ca^+2^/Mg^+2^ salts supplemented with 1 mM sodium pyruvate and 10 mM HEPES, pH 7.4. On ice, individual cells were isolated by titurating 10 times using a glass pasture pipette with the tip barely fire polished. We adjusted the volume to 1.5 mL, by adding 1 mL of HBSS with Ca^+2^/Mg^+2^ salts + Na pyruvate + HEPES, restoring the divalent cations by adding HBSS so that the nondispersed tissue could settle for 5 min, on ice. In the tissue culture laminar hood, we transferred the supernatant into a new 15 mL tube and centrifuged for 1 min at 900 rpm, 4°C. We gently resuspended the pellet in 2 mL of HBSS with Ca^+2^/Mg^+2^ salts + Na pyruvate + HEPES and took an aliquot for counting (2 mL for approx. 5 embryos). We then plated ~ 1 × 10^5^ cells/well of a 24-well or 2 × 10^5^/in 12-well plates. Each set of plates were coated with poly D-lysine containing poly L-lysine coverslips for micro immunocytochemical confirmation of neuronal integrity.

### 2.3. Bioinformatics Methods

 The  *α*-synuclein RNA sequences were selected using the NCBI Gene search and the Ensembl database (see [34]). Since the 5′ UTRs were of primary interest, the coding sequences (CDS) were mostly disregarded, apart from the initiating AUG. As we reported [34], the splice junction between two exons occurred at a CAGUGU site 25 = nucleotides from the AUG and that this pattern was conserved among the other species investigated. Thus, in order to study a balanced sequence, 25 nucleotides before the splice junction from the first exon were used to create 50 nucleotide RNA sequences (mouse and rat had 52 nt sequences due to insertions).

The ClustalX2 graphical program was used to align the RNA sequences to identify any evolutionary conservation between species. For alpha synuclein, the AUG start region of the CDS was a reference point for sequence alignments, allowing a comparison of the sequences in both exons around the splice junction, due to high local conservation fidelity. Secondary sequences were then generated by the University of Vienna's RNAfold webserver (with standard settings) and were annotated using the RNAfold command-line software. The RNAfold server provided the most probable secondary structure based on minimum-free energy calculations.

We calculated the alignment homology by comparing any species' RNA sequence (as listed) against the *Homo sapiens* sequence on each side of the splice junction. We also calculated the homology between the core L- and H-ferritin IREs with that of alpha synuclein and APP mRNAs. Only nucleotides that matched respective to the *Homo sapiens* sequence were scored a point; we determined the percent homology on each side by totaling the points scored and dividing by the total number of nucleotides on that side. The results from each side were then compared to illuminate the difference in conservation across the splice junction.

### 2.4. Constructs

 The *SNCA*-5′UTR-pGL3 construct was generated from the pGL3 expression vector (Promega). In this case, a PCR fragment encoding 48 base  *α*-synuclein 5′UTR was cloned between the Hind-III and Nco1 sites in front of start codon of the luciferase gene in PGL3. Transiently transfected cells expressed either pGL3 or the *SNCA*-5′UTR-pGL3 construct. Stably transfected neural cell lines were generated via neomycin selection after cotransfection with the RSV2-neomycin plasmid to express the *SNCA*-5′UTR-pGL3 construct (H2A cells) or pGL3 (control cells).

### 2.5. Stable-Transfection-Based Screen of a Library of Natural Product Inhibitors of *α*-Synuclein Translation

 A 720 compound natural products, including added phenserine and posiphen, from Microsource Discovery Systems, Inc, was screened at a concentration of 2 uM in triplicate for inhibition of luciferase expression using (APP cells) and (*SNCA *cells). A compound was scored as a hit if all replicates gave >65% inhibition in this assay, and as contradictory if at least one, but not all replicates gave >65% inhibition. In order to rule out compounds that reduced luciferase expression due to toxicity, the entire library was also screened in both cell lines for cytotoxicity, using alamar blue (Biosource, Inc.) as the readout. Percent inhibition in the alamar Blue assay was calculated and compared with percent inhibition of luciferase activity. Posiphen and phenserine were among the compounds for which the difference was greater than 40 and which scored as a hit or contradictory in the luciferase assay.

### 2.6. Transient Transfection Assays

After 24 hr transfection of SH-SY5Y cells in a 100 mm dish with either (i) *SNCA*-5′UTR-pGL3 or (ii) pGL3 parental vector, cells were then passaged into 6 wells and tested with posiphen and phenserine (15 *μ*M = for 48 hr) as shown in Figures 3 and 4 for which dose-response assays were also conducted (not shown) [34, 50].

### 2.7. Luciferase Assay

 Cells were plated at 2000 cells/well (APP) or 4000 cells/well (*SNCA*) in a 384-well white flat-bottom plate (Greiner) in a volume of 50 uL of media. Following overnight incubation, 50 nL of 5 *μ*M in DMSO were added using a VPN Pintool. Cells were returned to the incubator of 48 h, then assayed for luciferase activity. Plates were allowed to equilibrate to room temperature. After addition of 25 uL Steady-Glo reagent (Promega), plates were vortexed for 30 sec and 35 minutes later luminescence read on an Infinite F2000 plate reader (Tecan) [34, 50].

### 2.8. Western Blot Assay

 Human SH-SY5Y cells and primary cortical neurons (E18 fetal cells) were exposed to increasing concentrations of phenserine and posiphen for 48 hours. Cytoplasmic protein lysates were prepared by homogenizing the cells in midRIPA buffer (25 mM Tris pH 7.4, 1% NP40, 0.5% sodium deoxycholate, 15 mM NaCl, protease inhibitors, RNase inhibitor, and 10 *μ*M DTT). Western Blotting for alpha-synuclein was performed using mouse monoclonal anti-alpha-synuclein (BD Transduction Laboratories) and anti-beta-actin (Chemicon). The blots were developed using chemiluminescence (PIERCE) and visualized with a PhosphoImager (BioRad, Hercules, CA), and bands were quantified using QuantityOne software (BioRad).

## 3. Results and Discussion

**Figure 1:**
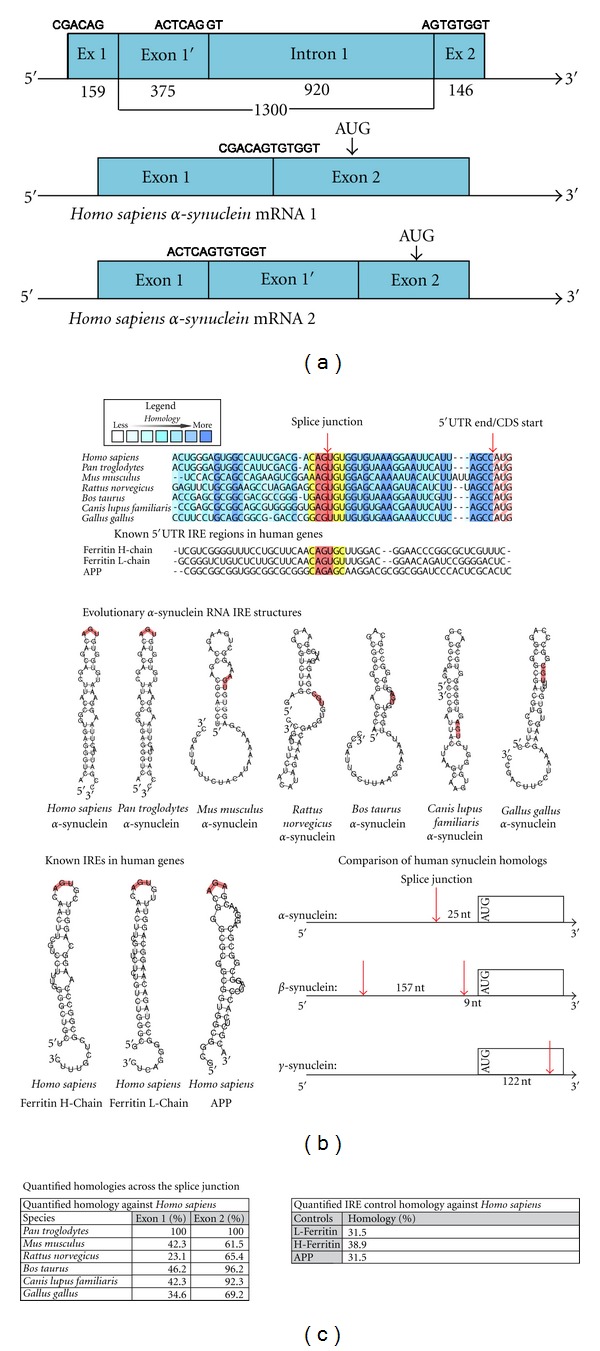
The 5′untranslated region (5′UTR) of the Parkinson's disease alpha-synuclein (*SNCA*) transcript is homologous to the iron-responsive element (IRE) in H-ferritin mRNA. (a) *Top panel*: The* SNCA* 5′UTR is encoded by exon-1 and exon-2 of the *SNCA* gene, which can be alternatively spliced to generate either a shorter exon-1/-2 transcript or a longer exon-1′-2 transcript [58]. *Bottom panel:* evolutionary alignment of the *SNCA* 5′UTR relative to the human sequence and the CAGUGN loop/splice site sequences [33]. We quantified the homology across the *SNCA* splice junction in the 5′untranslated region. (b) RNA/FOLD computer program predictions of RNA stem loops from *SNCA* 5′UTR sequences (Δ*G* = 53 kcal/mol). The human *SNCA* stem loop resembles the classical IRE RNA stem loop (5′CAGUGN3' loop motif) that controls iron-dependent L- and H-ferritin translation and transferrin receptor (TfR) mRNA stability. Stem loops from the 5′UTRs of several species were predicted to be folded, as described in the Materials and Methods section, and the pseudotriloop AGU is depicted in red lettering at the apex of the H-ferritin IRE [59] where the analogous AGA from the APP IRE is depicted [50]. The human *SNCA *mRNA exhibited an AGU triloop, whereas, in lower vertebrates, this AGU motif was located in the stem regions of these transcripts. Shown are the arrangement of splice sites and 5′UTR structures in the *SNCA*, *SNCB*, *SNCG* mRNAs. *Tabulated:* the first table shows the homology between the IRE-like domains in each exon of the human alpha-synuclein compared to its counterparts in different mammalian species. The second table shows the homology between the 5′untranslated regions of *SNCA *with the APP and ferritin L- and H-chains. This data underscored our identification of cotranslational repression of APP and alpha-synuclein by small molecules, such as phenserine and posiphen.

In Figure 1, we performed a full bioinformatic analysis of the* SNCA* 5′UTR demonstrating by computer-mediated predictions [50] that the 5′UTR of the *SNCA* transcript folds into a unique RNA stem loop resembling an iron-responsive element (IRE) RNA structure that is related to, but distinct from, the H-ferritin and APP 5′UTR-specific IREs [33]. We are currently testing the capacity of intracellular iron chelation with desferrioxamine to repress neural  *α-*synuclein translation acting via the IRE in the 5′UTR of its transcript, as we reported for the APP and ferritin mRNAs [42, 51].

Previously, the RNA-directed anticholinesterase drug phenserine, together with its cholinergically inert chiral (+) enantiomer, posiphen, which are both in clinical development for AD, was shown to therapeutically limit brain A*β* levels in wild-type and AD mouse models [46]. This action was mediated, in whole or in part, by lowering the rate of synthesis of APP, from which A*β* is proteolytically cleaved. Here, we sought to demonstrate that phenserine and posiphen, likewise, blocked  *α-*synuclein expression via their related 5′UTRs, encoding variant versions of the iron-responsive element RNA elements that potentially bind iron-regulatory proteins.

**Figure 2:**
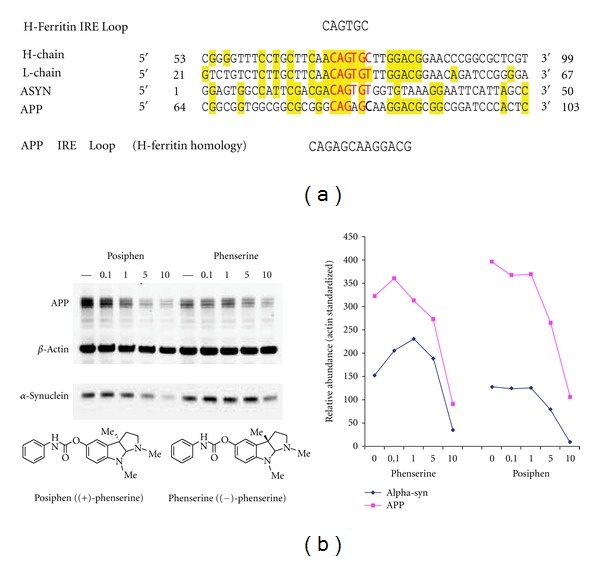
Posiphen and phenserine decrease both APP and  *α-*synuclein levels dose-dependently in dopaminergic SH-SY5Y cells. Panel A: the 5′UTRs of both the *APP *and *SNCA *genes are 50% homologous with the IRE H-ferritin mRNA. Panel B: SH-SY5Y cells were treated with concentrations ranging from 0 to 10 *μ*M phenserine and posiphen for 48 h. Harvested cell lysates were prepared. Quantitative Western Blotting established the anti-*α-*syn efficacy of posiphen and phenserine (IC_50_ < 5 *μ*M); after standardization for *β*-actin (Densitometry of multiple lanes (*n* = 8) by ImageQuant). Cell viability was unaffected (measured by standardized ATP levels/cell (Tm (Cell-Titer-glo, Promega, Inc.))).

**Figure 3:**
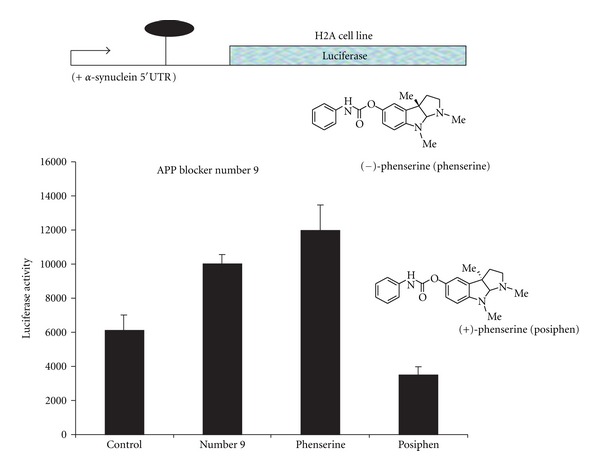
Posiphen is a stereospecific inhibitor of alpha synuclein 5′UTR-directed translation. Transient transfection assays with the *SNCA*-5′UTR-pGL3 construct (H2A cell line). Posiphen was a highly selective inhibitor (10 *μ*M) of  *α*-synuclein 5′UTR-driven expression of a luciferase reporter gene. This stereoisomer of phenserine inhibited  *α*-synuclein 5′UTR-driven luciferase expression in neural cells (*SNCA* 5′UTR-positive transfected neural cells (*N* = 7)). By contrast, phenserine and the known APP-specific translation number 9 blocker did not suppress alpha synuclein 5′UTR-conferred translation in H2A cells. Phenserine and APP blocker number 9 increased *SNCA* 5′UTR-conferred translation. These data support the mechanism-of-action of posiphen as a highly selective blocker of alpha synuclein 5′UTR activity, whereas phenserine (same chemical structure but stereoisomer of posiphen) was previously shown selectively inhibiting translation driven by the APP 5′UTR.

**Figure 4:**
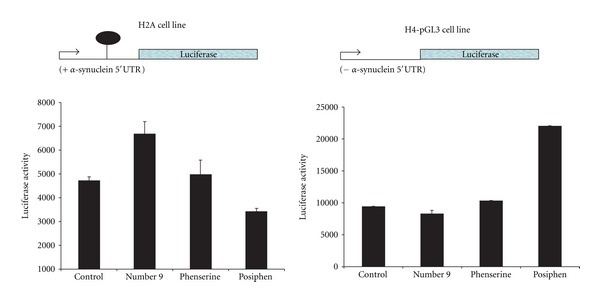
Selectivity of posiphen to inhibit translation driven by alpha-synuclein 5′UTR sequences: a second set of transient transfection experiments in which posiphen (10 *μ*M) selectively inhibited alpha-synuclein 5′UTR-conferred luciferase expression in SH-SY5Y neural cells (*SNCA* 5′UTR-positive transfectants, (*N* = 6)). Confirming selectivity, posiphen increased luciferase expression in pGL3-transfected SH-SY5Y cells (**pGL3-SH-SY5Y serves as an experimental control since these cells were transfected with pGL3, which is the same as the H2A construct but lacks the  *α*-synuclein 5′UTR).

 To elucidate whether our defined target would translate across species, we provide the results of an evolutionary evaluation of the conservation of IRE RNA stem loops in *SNCA* mRNA as shown in Figure 1. This is the RNA secondary structure sequence, together with the APP 5′UTR, that is targeted by phenserine and posiphen, as shown in Figures 2–4. The alpha-synuclein-specific IRE stem loop was formed at the splice junction of the first two exons in *SNCA* gene [35]. We also compared the predicted structure of the *SNCA* mRNA IRE with the canonical IREs in the H-ferritin and APP transcripts, which are transcribed from the single first exon of their genes confirming the uniqueness of translational repression of *SNCA* mRNA via its 5′UTR.

The anticholinesterase phenserine and its (+) enantiomer, posiphen, are proven APP 5′UTR mediated drugs with known pharmacokinetics in rodents and humans and identified target concentrations [46, 52]. Since we anticipated that both agents would be active in limiting  *α-*synuclein translation via its 5′UTR, we had spiked an FDA library with posiphen and phenserine when we formerly screened against the* SNCA* 5′UTR RNA target [34]. Here, we confirm that both posiphen and phenserine repressed the *SNCA* 5′UTR-driven translation of a luciferase reporter in stable cells lines. Their capacity to inhibit *SNCA* mRNA translation is similar to that of certain other defined FDA drug leads, including three glycosides and an immunosuppressant, mycophenolic acid (secondary Fe chelator), as we previously reported [34].

In Figure 2(a), the 5′UTRs of both APP and *SNCA *showed 40% homology to each other and also 50% homology to the IRE in H-ferritin mRNA. Multiple Western blot experiments were hence conducted to determine the impact of phenserine compared to posiphen to limit  *α-*syn compared to APP expression. In this regard, SH-SY5Y cells were treated with both compounds for 48 hours with concentrations ranging from 0 to 10 *μ*M. Viability studies determined that this range was well tolerated, in accordance with prior studies. In general and as shown in Figure 2(b), posiphen (in addition to but potentially slightly more potently than phenserine) decreased levels of  *α-*syn in a dose-dependent manner in cultured neural cells (SH-SY5Y) as previously reported for APP. Whether this higher potency would translate to primary neurons and *in vivo* is a focus of future studies. In this paper, this was achieved with a preliminary determined IC_50_ in the order of 5 *μ*M, in the absence of toxicity.

Multiple transient transfections of SH-SY5Y cells were performed with the constructs that either translated luciferase driven by the 48 base SNCA 5′UTR (*SNCA*-5′UTR-pGL3) or the empty pGL3 expression vector (Figures 3 and 4). As shown (Figures 3 and 4), posiphen 50% repressed* SNCA* 5′UTR-conferred translation of a luciferase reporter transcript. In this regard, posiphen proved a highly selective inhibitor of *SNCA* 5′UTR driven activity since this chirally pure compound inhibited *SNCA* 5′UTR-driven luciferase expression in H2A neural cells (i.e., *SNCA* 5′UTR-positive stably transfected neural cells). By contrast, phenserine and the known APP translation blocker, compound number 9 (included as a comparator), did not suppress *SNCA* 5′UTR conferred translation in H2A cells. Indeed, phenserine and compound number 9 elevated *SNCA* 5′UTR-conferred translation.

These data support the mechanism-of-action of posiphen as a highly selective blocker of *SNCA* 5′UTR activity. However, phenserine—with the identical chemical structure but in a different three-dimensional (chiral) configuration—that has previously been shown to effectively inhibit translation driven by the APP 5′UTR clearly has different actions to posiphen at the *SNCA* 5′UTR. From this, we can deduce that the element of the *SNCA* 5′UTR targeted by posiphen has a stereospecific component. Additionally, since phenserine lowers  *α-*syn levels (Figure 2(b)), further *SNCA* RNA sequences are likely involved in controlling this pathway of  *α-*syn translational regulation.

 Extending beyond transformed neuronal cell lines, primary neurons from *wild-type mice* and PAC *SNCA* transgenic mice (PAC-Tg *SNCA*) were evaluated for the capacity of posiphen to repress  *α-*syn expression, as shown in Figure 6 (tested at 100 nM drug concentration). Posiphen proved not only active and well tolerated in SH-SY5Y cells but consistently reduced human  *α-*synuclein expression in primary neurons (E18) at doses as low as 1 uM (75% reduction, not shown) without toxicity. This margin appeared to be greater than its capacity to lower APP production (20%) (utilized as a positive control) in these same cells (data not shown).

**Figure 5:**
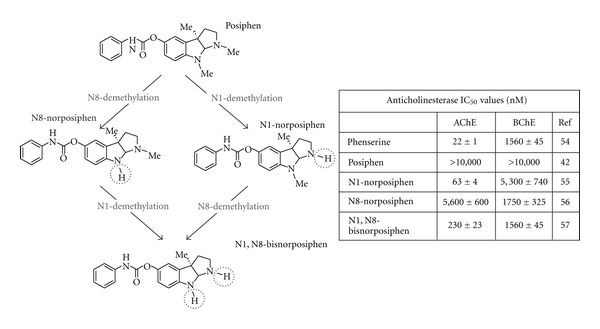
Metabolic analogs of posiphen and their respective anticholinesterase activities [60]. Posiphen is devoid of anticholinesterase activity. However, its phase 1 metabolites, N8 demethylated, N1 demethylated, and di-demethylated N1, N8-bisnorposiphen showed *ex vivo* AChE and BChE inhibitory activity of clinical relevance [61, 62]. The compound N8-bisnorposiphen demonstrated no AChE activity. This activity has proven to be dose limiting in human safety studies.

Following oral administration of posiphen to rodents, dogs, and human, the compound is subjected to metabolic processes and generates the same metabolic profile across these species. Specifically, via a phase 1 metabolism, posiphen undergoes N-demethylation in both the N1 and N8 positions to generate the respective primary metabolites, N1-norposiphen and N8-norposiphen (Figure 5). Each then undergoes further N-demethylation to generate the common metabolite, N1, N8-bisnorposiphen. Unlike phenserine, posiphen is devoid of cholinesterase inhibitory activity and, therefore, can be advantageously administered at higher clinical doses (in the order of 5, to 8-fold greater).

**Figure 6:**
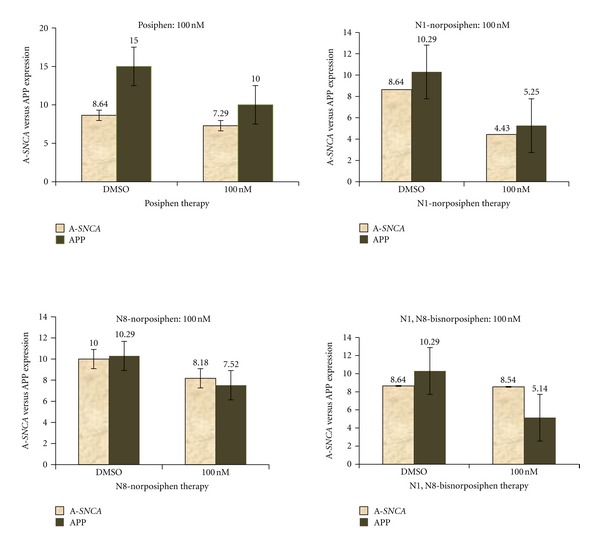
Percent inhibition of  *α-*synuclein/APP by posiphen compared with its metabolic analogs in primary E18 neurons (from PAC-Tg(*SNCA*) mice). Densitometry and quantitation of the relative levels  *α-*synuclei*n*/APP from western blots (*β*-actin standardized) after treating primary neurons with posiphen and each of its three metabolic analogs at 100 nM concentration for 48 hours.

**Figure 7:**
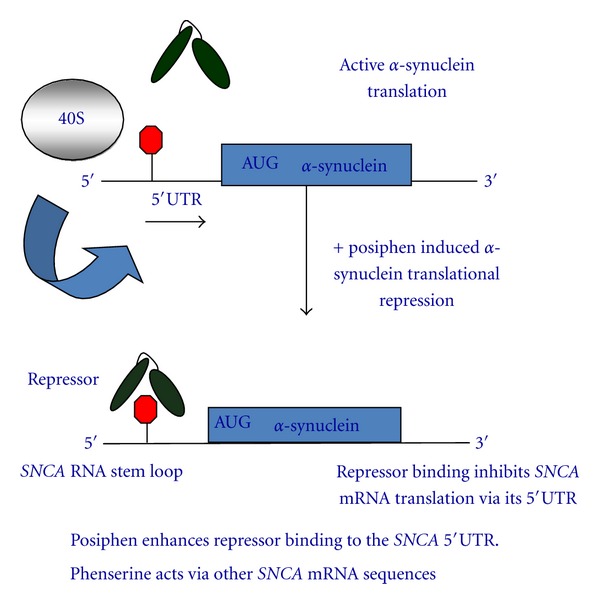
A model for translation repression of alpha-synuclein in which its transcript interacts with an RNA repressor protein. In the classical translation repression model, Iron-regulatory protein-1 (IRP1) controls iron dependent expression of the ferritin L- and H-chains. By analogy, phenserine, posiphen and its metabolic analogs would be predicted to increase the repressor as an RNA binding protein to interact with the SNCA 5′UTR after drug treatment. This should prohibit 40S ribosome access to the 5′UTR of SNCA mRNA to lower translation.

As shown in Figure 5, specific N-demethylated metabolites (in particular the N1-nor and N1,N8-bisnorposiphen) possess potentially clinically relevant IC_50_ values to inhibit acetylcholinesterase (AChE). Such activity is less than phenserine and would be expected to have a slow onset in line with the time-dependent metabolism of posiphen to generate its metabolites. However, with regard to actions on  *α-*synuclein expression, activity of the metabolites at this target, where posiphen is potent, could usefully increase and extend the drug's *in vivo* efficacy. To elucidate this and aid planning for future animal studies, we characterized the relative capacities of posiphen's metabolic analogs to impact  *α*-syn expression (Figure 6).

Figure 6 shows a Western Blot analysis representative of three experiments that compared the efficacy of posiphen with that of its three primary metabolites to limit  *α*-synuclein expression *ex vivo* using E18 primary cortical neurons from human *SNCA* PAC mice. We consistently measured that *N1-norposiphen* (possessing AChE activity) proved most potent to limit  *α-*synuclein expression (by 50%), and, likewise, APP expression was also reduced. As assessed at 100 nM in Figure 6, the other metabolites proved to be less active at these targets, indicating that relatively small structural changes (i.e., the loss of a methyl moiety) have significant impact. An assessment of dose-response for each metabolite is a focus of future studies, particularly within the achievable clinical range of the agents in human and *in vivo *range in animal models (Dr. Maria Maccecchini and Dr. Robert Nussbaum, personal communications).

## 4. Conclusions

We previously reported that phenserine, a (-)-physostigmine analogue and anticholinesterase that reached phase III clinical assessment for AD [53–55], inhibited APP translation though its 5′UTR [56, 57]. Phenserine was found to be dose limiting consequent to its AChE inhibitory action, causing the classical cholinergic action of tremors in animal models and nausea in humans. Posiphen, the chirally pure (+) enantiomer of phenserine, by contrast possesses no direct anticholinesterase activity and, likewise, repressed neural AD-specific APP translation via its 5′UTR in mice. It has recently been developed (QR Pharma, Berwyn, PA) to the clinic as an APP synthesis inhibitor in AD to lower both brain A*β* generation as well as the levels of other toxic proteolytic products of APP. With a very different pharmacological and pharmacokinetic profile to phenserine, it has recently completed single-and multiple-dose escalating phase I clinical assessment in humans, appearing well-tolerated, and a proof of mechanism study, indicating target engagement.

In this paper, we demonstrated in human immortal neuronal cultures, and then *ex vivo* in primary cortical neurons from a human* SNCA* genomic mouse model, that *posiphen* acts as a safe 5′UTR-directed inhibitor of toxic  *α-*synuclein buildup. A final validation of the *SNCA* 5′UTR target is to be achieved by unchanged *β*-synuclein (*β-*syn) and  *γ*-synuclein (*γ-*syn) expression and is a focus of current studies. Importantly, we determined that key primary metabolites of posiphen, likewise, lower  *α-*syn expression and may hence add to posiphen's actions on this target in animal and human studies.

Of further interest, we established that phenserine similarly effectively reduced  *α-*syn levels but achieves this via a different mechanism, compared to posiphen, potentially by interacting through other elements in the 5′UTR or even 3′UTR, and thereby highlighting the sensitivity of the target to small structure-activity changes. These studies additionally demonstrate how  *α-*synuclein and Aß pathomechanisms can converge via interaction of the two amyloidogenic proteins [38–40], although Aß and  *α-*syn rarely co-localize in amyloid deposits [41], but provide evidence that a drug targeting one disease may have therapeutic potential in another.

 In this regard, we noted that there is 50% sequence similarity between the 5′ UTRs of the APP and *SNCA *genes. We therefore predicted overlap in the spectrum of drugs that would suppress APP mRNA translation through its 5′UTR with those that suppress  *α-*synuclein, highlighting in particular phenserine and posiphen (with preliminary IC_50_ values in the order of 5 *μ*M and less). In future studies, we aim to characterize the anti-*α-*synuclein efficacy of posiphen analogs in addition to metabolic analogs and other compounds in primary neurons and establish the *in vivo* efficacy of the most effective *SNCA* 5′UTR translation blockers.
